# Appendiceal abscess in a giant left-sided inguinoscrotal hernia: a rare case of Amyand hernia

**DOI:** 10.1186/s40064-015-1162-9

**Published:** 2015-07-26

**Authors:** Massimo Mongardini, Alessandro Maturo, Livia De Anna, Giada Livadoti, Valerio D’Orazi, Paolo Urciuoli, Filippo Custureri

**Affiliations:** Department of Surgical Sciences, “Sapienza” University of Rome, “Umberto I” University Hospital, Viale Regina Elena 324, 00161 Rome, Italy

**Keywords:** Giant inguinoscrotal hernia, Amyand hernia, Appendiceal abscess, Surgery

## Abstract

The hernia of Amyand is an inguinal hernia containing the appendix in the sac. It is a rare pathology often diagnosed only intra-operatively. We report a case even more rare of a giant left-sided inguinoscrotal Amyand hernia with appendiceal abscess without clinical findings of incarceration/strangulation, occlusion, perforation, or acute scrotum and with the presence in the sac of the caecum and other anatomical structures (last ileal loops, bladder and omentum). The 68-years-old man patient successfully underwent surgical treatment only through the hernia sac (meshless repair according to Postempski technique).

## Background

Inguinal hernia defined as the protrusion of an organ or fascia through the wall of the containing cavity is one of the most frequent surgical procedures that a surgeon faces. The giant inguinoscrotal hernia is defined as such when it extends beyond the midpoint of the thigh with the patient in a upright position (Hodgkinson and McIlrath 1984). These hernias are uncommon in developed countries and patients usually refuse to admit this kind of problem for years (Cavalli et al. 2014) until the hernia causes major discomfort, especially during walking and standing, reducing the quality of life. Giant hernias can be burdened with complications such as bowel obstruction and strangulation that need emergency surgery.

The contents of inguinal hernia sac differ from case to case. The presence of appendix in an inguinal hernia sac is called hernia of Amyand, in honor of the French military surgeon Claudius Amyand (1680–1740) who, exiled in England, first successfully carried out in 1735 an appendectomy in a 11 years old boy with fecal inguinal fistula (Gupta et al. 2005; Singh et al. 2011). The hernia of Amyand with normal appendix is a rare event (about 1% of all inguinal hernias) and even more rare is a hernia with appendicitis (0, 1%) (D’Alia et al. 2003; Singh et al. 2011; Morales-Cardenas et al. 2015). Amyand hernia is more common on the right side (Pellegrino and Feldman 1992; Maeda et al. 2014); on the left side it is associated with hypermobility of the caecum or situs viscerum inversus or malrotation of the gut (Breitenstein et al. 2005; Maeda et al. 2014). A possible etiopathogenic mechanism of appendicitis in Amyand’s hernia is the reduction of blood flow due to adhesions that make the hernia not reducible and/or the vascular compression exerted on the arteriosus vessels by external inguinal ring (Sengul et al. 2011).

The Amyand hernia has been classified into four types according to its severity: type 1, with normal appendix treated with reduction or appendectomy (depending on age) and hernioplasty with mesh; type 2, with acute appendicitis without peritonitis, treated with appendectomy by an inguinal access and hernioplasty without mesh; type 3, with appendicitis and peritonitis treated with laparotomy appendectomy and hernioplasty without mesh; and type 4, with appendicitis, appendiceal mucocele or cancer and/or others abdominal comorbidities in which the most appropriate surgical treatment must be evaluated case by case (Losanoff and Basson 2008).

The Amyand hernia with normal appendix is a rare event and even more rare is a hernia with appendicitis often diagnosed only intra-operatively. We present here a case even more rare of a giant left-sided inguinoscrotal Amyand hernia with appendiceal abscess and the presence in the sac of a tract of the ascending colon, last ileal loops, a portion of bladder and omentum. This case did not present clinical findings of incarceration/strangulation, occlusion, perforation, or acute scrotum. The patient underwent successful surgical treatment only through the hernia sac with meshless repair according to Postempski technique.

## Case description

A 68-years-old man who at the age of 53 years underwent transurethral resection of the prostate (TURP) for adenomyomatosis, presented in our Department of Surgical Sciences, “Umberto I” Hospital, in Rome. He had been suffering for about 10 years for an increasing left-sided inguinoscrotal hernia becoming in the last week not more reducible. Inguinal scrotal pain and septic fever appeared 30 days before the admission to our Department. Suspecting urinary infection, his clinician had prescribed antibiotic therapy which proved partially effective.

At the admission the patient was in fair general conditions without vomiting. The left emiscrotum, covered with normal skin, appeared fully occupied by hernia that extended over the middle third of the thigh. The penis was not visible. The hernia (20 × 20 cm in latero-lateral and antero-posterior diameters) was of increased consistence and not reducible. The testis was not palpable. The abdomen was treatable, without distention, but with minimal peristalsis and bowel constipated but open to gas.

**Fig. 1:**
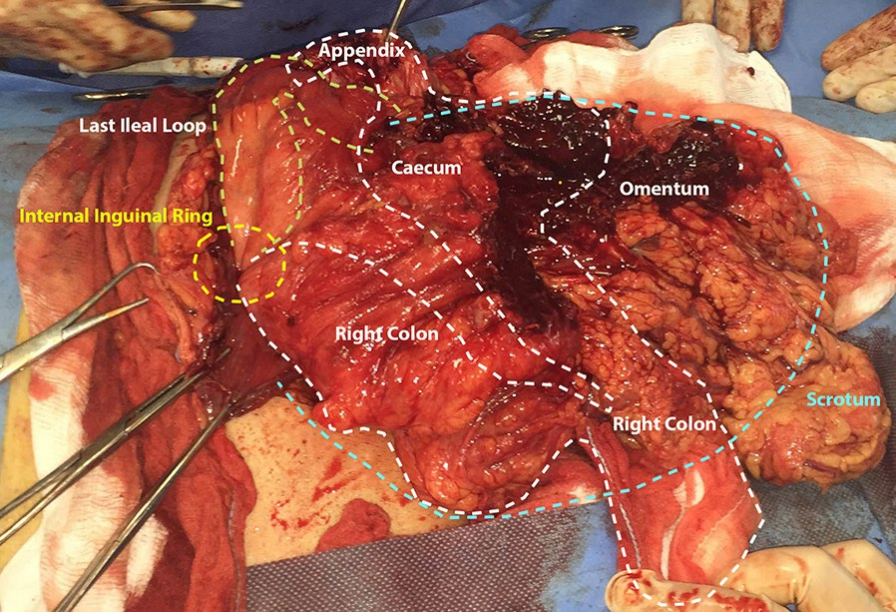
Intraoperative view after incision of the hernia sac in the scrotum.

**Fig. 2:**
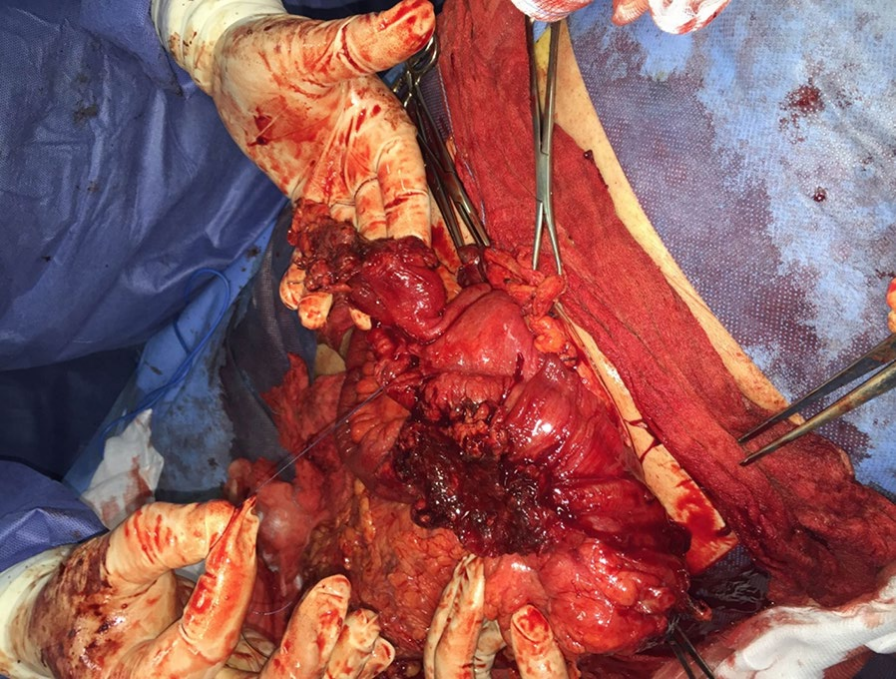
Perforated appendix.

**Fig. 3:**
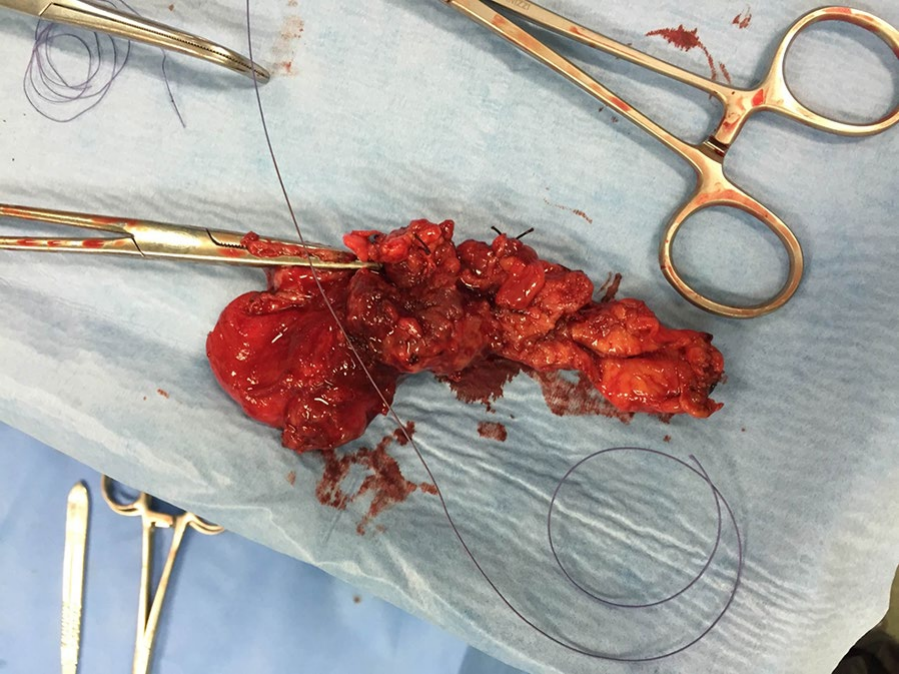
Specimen (appendix).

The patient underwent elective surgical treatment with an oblique incision (about 15 cm) parallel to the inguinal ligament prolonged up to the scrotum. Opening the hernial sac, the fibro-necrotic omentum was evident. It covered a huge abscess with thick fibrotic pseudocapsule and wide necrotic areas (Fig. 1). Once drained the abscess, the phlegmonous perforated appendix (Fig. 2), the caecum, the ascending colon, the last ileal loops and the bladder were debrided. After accurate debridement of the herniated organs, we proceeded with the appendectomy and resection of necrotic omentum with handmade sutures. Then we isolated the funiculus spermaticus and the testis from the other herniated viscera. Once checked the vitality of the herniated organs, we reduced them into peritoneal cavity. We positioned a transabdominal drainage on the finger guide introduced through internal inguinal ring. Finally, we performed a hernioplasty according to Postempski technique without mesh, considering the risk of infection. Histological examination confirmed the appendiceal phlegmon (Fig. 3) and the necrosis of the omentum removed (20 × 20 × 5 cm).

The postoperative course was regular. On the third postoperative (p.o.) day the drainage was removed. On the seventh p.o. day, after doing an abdominal ultrasound that showed no postoperative complications, the patient was discharged with the advice of wearing a restraining underwear and of avoiding physical exercises for about 6 months.

## Discussion

The hernia of Amyand with normal appendix is a rare event (about 1% of all inguinal hernias) and even more rare is a hernia with appendicitis (0, 1%) (D’Alia et al. 2003; Singh et al. 2011). Amyand hernia is more common on the right side (Pellegrino and Feldman 1992; Maeda et al. 2014); on the left side it is associated with hypermobility of the caecum or situs viscerum inversus or malrotation of the gut (Breitenstein et al. 2005; Maeda et al. 2014).

According to Losanoff and Basson that classified the Amyand hernia into four types based on its severity (Losanoff and Basson 2008), in our patient the hernia was classified of type 4. The risk of infection of the mesh could increase in case of partial resection of the colon or, as in our case, of the omentum (Hutchinson 1993; Monestiroli et al. 2007). In our patient general conditions and local examination were partly altered by antibiotic therapy. Therefore we diagnosed a simple giant hernia to be treated in election although not reducible. We have not considered necessary preoperative radiological investigations such as ultrasound or CT, although the latter is considered diagnostic for Amyand hernia (Luchs et al. 2000; Monestiroli et al. 2007).

Another important issue in the giant hernias is the increase of intra-abdominal and intra-thoracic pressure that follows the reduction of the contents into the peritoneal cavity. The increased pressure can cause, in severe cases, a respiratory or cardiac failure with compartmental syndrome resulting into worse morbidity and mortality rates (Kyle et al. 1990; Mehendal et al. 2000). In addition, closing the abdominal wall under tension is linked with a greater risk of wound dehiscence; in fact, 30% of patients who undergo such procedures suffer from hernia recurrence and/or wound dehiscence (Stoppa 1989). Recently some authors proposed a progressive preoperative pneumoperitoneum preparation, i.e. Goni Moreno protocol (Sabbagh et al. 2012), and/or elective resection of the herniated abdominal viscera (colon, ileum or omentum) to reduce endocavitary pressure (Patsas et al. 2010; Cavalli et al. 2014). The choice of the surgical access is still now discussed. The surgical incisions are different, depending on the severity of the clinical status: pararectal  enlarged to the scrotum (Cavalli et al. 2014), median laparotomy or inguinal incision prolonged to the scrotum should be considered (Ek et al. 2006; Valliattu and Kingsnorth 2008). In our patient the prolonged inguinal incision has allowed us to completely isolate the sac, to resect the appendix and the omentum, to reduce the hernia and to perform the reconstructive surgery.

To the best of our knowledge, it is impossible to establish the cases of Amyand hernia that led to appendiceal abscess (Al-Mayoof and Al-Ani 2014; Michalinos et al. 2014) and the present report is certainly one of these very rare cases. In agreement with other findings (Karanikas et al. 2015), we believe that, owing to the rarity of Amyand hernia and the wide variance of its clinical characteristics, every case provides useful information toward the treatment of this type of hernia until standardization of treatment is achieved.

## Conclusions

Inguinal hernia is one of the most frequent surgical procedures that a surgeon faces and Amyand hernia is a rare event whose management involves a laborious surgical technique. The surgical strategy that we performed (inguinoscrotal incision, abscess drainage, accurate debridment, appendectomy and omentectomy, open repair without mesh) was the best treatment for an exceptional case of Aymand hernia. The patient recovered uneventfully and was discharged home on postoperative day 7. At control at 30 days the patient was asymptomatic. He claimed to be satisfied with the treatment received and that had returned to normal activities.

In conclusion we would like to highlight the rarity of the case reported and the technical issues related to the presence in the hernia sac of huge appendiceal abscess, other viscera and necrotic areas. Also we stressed the success of our surgical approach achieved only through the hernia sac, by a simple inguinal-scrotal incision and with the placement of a transabdominal drainage positioned on the finger guide introduced through internal inguinal ring. We believe that our case may contribute to Amyand hernia surgical treatment standardization. Anyway the surgeon should be ready to tailor the surgical strategy by customizing the procedure to the single patient.
